# Differential Cellular Stiffness Contributes to Tissue Elongation on an Expanding Surface

**DOI:** 10.3389/fcell.2022.864135

**Published:** 2022-03-29

**Authors:** Hiroshi Koyama, Makoto Suzuki, Naoko Yasue, Hiroshi Sasaki, Naoto Ueno, Toshihiko Fujimori

**Affiliations:** ^1^ Division of Embryology, National Institute for Basic Biology (Div. Embryology, NIBB), Okazaki, Japan; ^2^ Department of Basic Biology, School of Life Science, SOKENDAI (The Graduate University for Advanced Studies), Hayama, Japan; ^3^ Division of Morphogenesis, National Institute for Basic Biology (Div. Morphogenesis, NIBB), Okazaki, Japan; ^4^ Amphibian Research Center, Graduate School of Integrated Sciences for Life, Hiroshima University (ARC, Hiroshima Univ.), Higashihiroshima, Japan; ^5^ Laboratory for Embryogenesis, Graduate School of Frontier Biosciences, Osaka University (FBS, Osaka Univ.), Suita, Japan

**Keywords:** pattern formation, morphogenesis, tissue elongation, cellular stiffness, vertex model, theory, mouse notochord

## Abstract

Pattern formation and morphogenesis of cell populations is essential for successful embryogenesis. Steinberg proposed the differential adhesion hypothesis, and differences in cell–cell adhesion and interfacial tension have proven to be critical for cell sorting. Standard theoretical models such as the vertex model consider not only cell–cell adhesion/tension but also area elasticity of apical cell surfaces and viscous friction forces. However, the potential contributions of the latter two parameters to pattern formation and morphogenesis remain to be determined. In this theoretical study, we analyzed the effect of both area elasticity and the coefficient of friction on pattern formation and morphogenesis. We assumed the presence of two cell populations, one population of which is surrounded by the other. Both populations were placed on the surface of a uniformly expanding environment analogous to growing embryos, in which friction forces are exerted between cell populations and their expanding environment. When the area elasticity or friction coefficient in the cell cluster was increased relative to that of the surrounding cell population, the cell cluster was elongated. In comparison with experimental observations, elongation of the notochord in mice is consistent with the hypothesis based on the difference in area elasticity but not the difference in friction coefficient. Because area elasticity is an index of cellular stiffness, we propose that differential cellular stiffness may contribute to tissue elongation within an expanding environment.

## 1 Introduction

Pattern formation and morphogenesis by cell populations includes cell sorting, intermixing of different cell types, etc. These patterns are observed in various embryos and tissues such as germ layers, oviduct, and cochlea (Yamanaka and Honda, 1990; Steinberg, 2007; Krieg et al., 2008; Togashi et al., 2011). A few hypotheses have been proposed to explain these phenomena, including the differential adhesion hypothesis by Steinberg (Steinberg, 1963) in 1963. According to these hypotheses, either differential cell–cell adhesion or cell–cell interfacial tensions are considered, and their strengths are assumed to differ among cell types. The roles of these mechanical parameters in tissue organization have been demonstrated by both theoretical and experimental studies (Duguay et al., 2003; Krieg et al., 2008; Manning et al., 2010).

There are cellular mechanical parameters other than cell–cell adhesion forces and cell–cell interfacial tensions as follows, but the roles of these parameters in tissue organization have not been investigated. Area elasticity of each cell and coefficient of viscous friction forces are generally considered as basic parameters in theoretical studies where the vertex model and the Cellular Potts model are often used as standard multicellular models (Zajac et al., 2003; Fletcher et al., 2014). These parameters are critical for describing cellular and tissue behaviors. Area elasticity denotes resistance against changes in apical cell surface area. For instance, when the apical cell surface is either stretched or compressed by external mechanical forces, the apical surface either increases or decreases, respectively, and the extent of the area change is determined by both the strength of the external forces and the area elasticity. Therefore, area elasticity can be considered as an index of cellular stiffness. Without this parameter, cells cannot maintain their apical surface area, and hence, this parameter is essential for theoretically describing epithelial cells. On the other hand, coefficient of viscous friction forces is derived from viscous friction forces exerted between cells and surrounding medium or tissues (Okuda et al., 2014); increased friction forces restrict both cell movement and deformation. The friction forces between cells and surrounding tissues are affected by cell–cell interactions, cell–extracellular matrix interactions mediated by focal adhesions, etc. (Smutny et al., 2017; Trepat and Sahai, 2018; Münster et al., 2019). A spatial difference in friction forces is involved in the positioning cell populations in fish embryogenesis (Smutny et al., 2017). However, the contributions of these two parameters to pattern formation and morphogenesis remain almost unknown.

**FIGURE 1:**
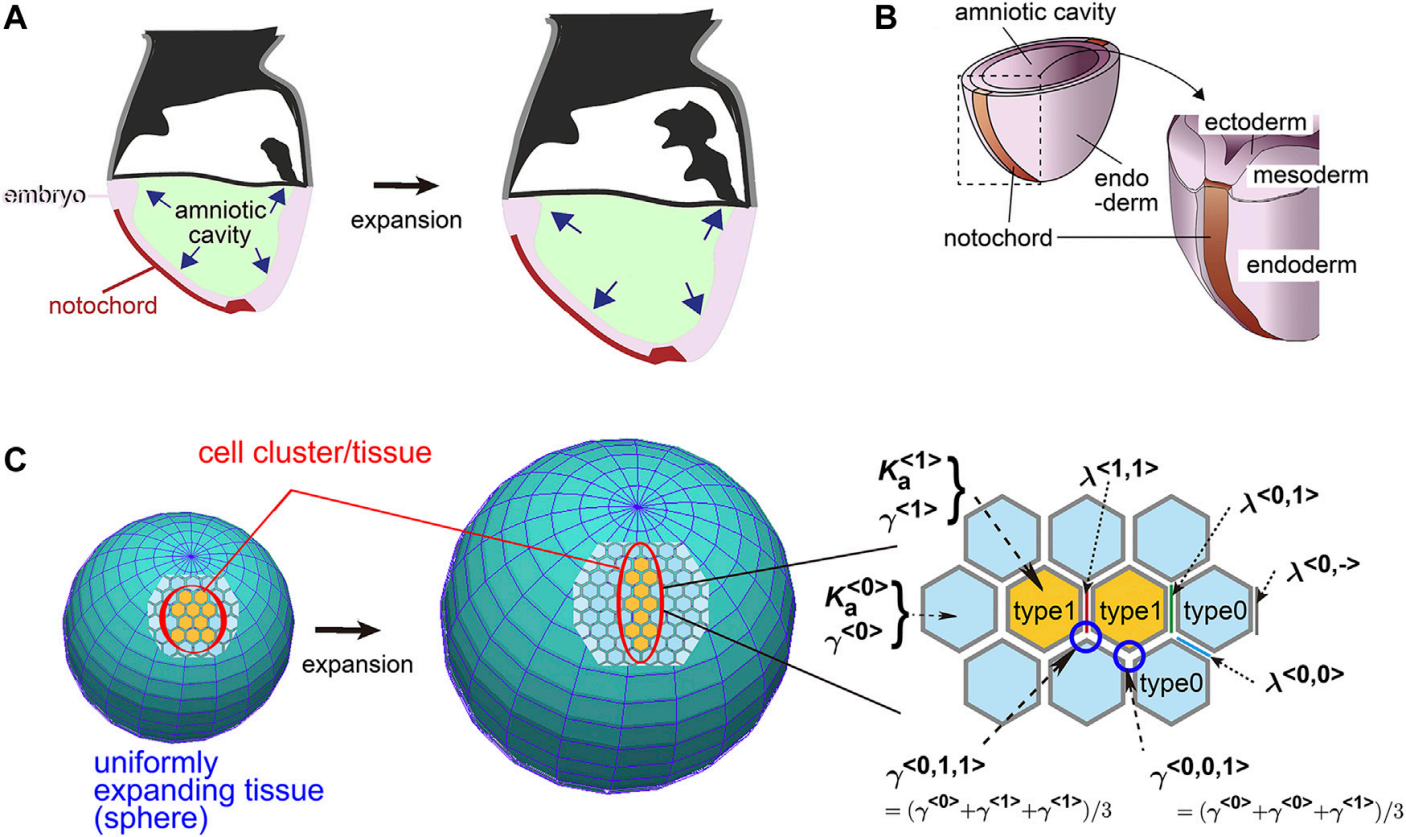
Mouse notochord, uniformly expanding field, and vertex model. **(A)** Cross–sectional illustration of a mouse embryo is shown. The amniotic cavity pushes the embryo on the surface of which the notochord is located. **(B)** Three–dimensional illustration of a mouse embryo with the notochord, the cell layers, and the cavity is shown. **(C)** A theoretical framework of a uniformly expanding field and vertex model is described. In the left panel, on a uniformly expanding tissue (two spheres before and after expansion), a cell cluster (orange) with its surrounding cells (light blue) is located. In the right panel, the vertex model is described where the area elasticities, the friction coefficients, and the line tensions are shown. The type 1 cells, orange; the type 0 cells, light blue. The panels A and B are originated from our previous study with permission (Imuta et al., 2014).

In this study, to elucidate the contributions of the above two parameters (i.e., area elasticity and coefficient of friction forces), we focus on a following theoretical model which we previously developed for describing elongation of the mouse notochord. The notochord is located on a surface of the mouse embryo (Figure 1A). The embryo around this developmental stage (embryonic days 5.5–8.5) is cylindrical or spherical in shape, at the central region of which there are amniotic cavities. The cavities increase their volumes (Figure 1A), and pushes the surrounding cell layers that are composed of ectoderm, resulting in expansion of the ectodermal layer (Figure 1B). This expansion is subsequently transduced to the outer cell layers, namely the mesoderm, endoderm, and notochord (Figure 1B). The outermost layers in the mouse embryos are composed of the endoderm and notochord during the early stages of notochord formation (Figure 1B). Therefore, the endoderm and the notochord would experience friction forces from expanding inner cell layers or the basement membranes between those layers. Previously, we reported that, by experimentally inhibiting the increase in the cavity’s volume, elongation of the notochord was inhibited, indicating that the expansion of the cavities promotes the notochord elongation (Imuta et al., 2014). Subsequently, we reported that in theory, on a uniformly expanding surface (Figure 1C, an expanding sphere), a cell cluster is elongated (Figure 1C, a cell cluster in orange), whereas friction forces are considered between the cells and the expanding surface (Koyama and Fujimori, 2020). This kind of expanding surface or field is analogous to an expanding rubber balloon in that the rubber membrane expands due to an increase in the volume of enclosed air. Importantly, even if the expansion is uniform or isotropic and a cell cluster has no intrinsic activity for directional migration, the cell cluster is elongated (Koyama and Fujimori, 2020).

In the above theoretical work, we assumed an isolated cell cluster corresponding to the notochord. But in the real tissues, the mouse notochord is surrounded by endodermal cell populations, resulting in a continuous epithelial cell sheet on the growing embryo. In the presence of two different cell populations, we have not theoretically demonstrated whether a cell cluster of interest can elongate, and what kinds of differences in mechanical parameters between these two populations are critical for elongation.

In this study, we assumed a second cell population corresponding to the endoderm (Figure 1C, type 0) that surrounds a cell cluster of interest corresponding to the notochord (Figure 1C, type 1), and determined theoretically if the cell cluster can be elongated. Our model is based on a vertex model where area elasticity, friction coefficient, and cell–cell interfacial tensions were considered as well as a uniformly expanding field. We found that the cell cluster can elongate, when either the area elasticity or the friction coefficient in the cell cluster is higher than that in the surrounding cell population. Moreover, when comparing theoretical outcomes and experimental observations in the mouse notochord, the elongation of the mouse notochord is consistent with a difference in area elasticity, but not in friction coefficient.

## 2 Theoretical Model

### 2.1 Vertex Model

We adopted the simplest two-dimensional vertex model in which the mechanical potential energy (*U*) of a system is provided by line tensions of cell–cell interfaces and the area elasticity of each cell. A cell cluster of interest is defined as type 1 cells, and the surrounding cell populations as type 0 (Figure 1C). The potential energy is defined as follows (Koyama and Fujimori, 2020):

$$U=\sum_{<k,l>}\lambda L^{<k,l>}+\sum_{n}\frac{1}{2}K_{\text{a}}^{<c>}{\left(\frac{a_{n}}{a_{\text{O}}}-1\right)}^{2}a_{\text{O}}$$ (1)

where λ and $L^{<k,l>}$ are the line tension and the length of the cell–cell interfaces between adjacent vertices *k* and *l* (Figure 1C), respectively. λ can have different values according to two cells sharing the cell–cell interface <*k*, *l*>. For instance, $\lambda =\lambda^{<0,1>}$ for the case that the two cells are type 0 and type 1, and $\lambda =\lambda^{<1,1>}$ for the case that the two cells are both type 1s (Figure 1C). $a_{n}$ is the area of the *n*th cell. $a_{\text{O}}$ and $K_{\text{a}}^{<c>}$ are a preferred area of the cell and the coefficient of area elasticity of type <*c*> cells, respectively (Figure 1C; $K_{\text{a}}^{<0>}$ and $K_{\text{a}}^{<1>}$ ). The preferred area is an apical surface area of a cell which is released from external forces; such cell is under a mechanically relaxed state. The coefficient of area elasticity functions to resist against changes in apical surface area from external forces. Therefore, this elasticity contributes to cellular stiffness. If the value of $K_{\text{a}}^{<c>}$ is large, the cell tends to retain its apical surface area around $a_{\text{O}}$ . Because cellular stiffnesses are different among cell types *in vivo* (Zhou et al., 2009; Swift et al., 2013), $K_{\text{a}}^{<c>}$ may be a cell type–specific property.

The force (*F*_*h*_) exerted on an *h*th vertex is calculated as follows:

$$F_{h}=-\nabla U$$ (2)

where ∇ is the nabla vector differential operator at each vertex. The motion of each vertex in polygonal cells is damped by friction forces and is described as follows:

$$V_{h}=F_{h}/\gamma_{\text{V}}$$ (3)

where *V*_*h*_ is the velocity of the *h*th vertex, and $\gamma_{\text{V}}$ is the coefficient of the friction of a vertex. $\gamma_{\text{V}}$ can have different values according to three cells sharing the vertex. For instance, $\gamma_{\text{V}}=\gamma^{<0,0,1>}$ for the case that the three cells are type 0, type 0, and type 1, and $\gamma_{\text{V}}=\gamma^{<0,1,1>}$ for the case that the three cells are type 0, type 1, and type 1 (Figure 1C). The friction coefficients for each cell type are defined as $\gamma^{<0>}$ and $\gamma^{<1>}$ for type 0 and type 1 cells, respectively. The coefficient for each vertex was defined as the mean of the coefficients for the three cells: e.g. $\gamma^{<0,0,1>}=\left(\gamma^{<0>}+\gamma^{<0>}+\gamma^{<1>}\right)/3$ (Figure 1C). Okuda et al. (2014) also assumed differential coefficients of friction. The friction is exerted between the cells and their surrounding medium or tissues. In this study, we assumed that the friction from the expanding field as defined below is dominant to that from the medium.

### 2.2 A Uniformly Expanding Field

A uniformly expanding field is analogous to the surface of an expanding rubber balloon; the area of the surface increases uniformly regardless of location on the surface. In the case of mouse early embryos around embryonic day 7.5, the volume of the inner cavity (e.g., the amniotic cavity) is increased, and thus the surface of the embryo is expanding. Cell proliferation within the surface tissue also causes it to expand (Koyama and Fujimori, 2020). We adopted a simplifying assumption that a field expands in two dimensions. In this study, we placed a cell cluster on the expanding field with a surrounding cell population.

We previously defined the modeling of a uniformly expanding field (Koyama and Fujimori, 2020). Briefly, when we arbitrarily defined a point on the two-dimensional field as an origin, other points are assumed to move away from the origin with speeds proportional to the distances between the points and the origin: $V_{\text{e}}\propto D_{\text{e}}$ , where *D*_e_ is the distance between the point and the origin, and *V*_e_ is the velocity. An object placed on this field also moves by *V*_e_ if there are no other forces. Consequently, the equation that describes the motion of each vertex is modified as follows:

$$V_{h}=F_{h}/\gamma_{\text{V}}+V_{\text{e}}$$ (4)

A similar formulation was previously proposed (Okuda et al., 2014). We can interpret this equation as follows: $V_{h}=F_{h}/\gamma_{\text{V}}+F_{\text{e|}h}/\gamma_{\text{V}}$ , where *F*_e|*h*_ is an apparent friction force provided by the expanding field. Under this assumption, we verified computationally and analytically that this expanding field does not yield any biased forces toward the cell cluster. Additionally, the expansion rate of the field was assumed to be temporally constant: *V*_e_ = *ε D*_e_, where *ε* is the expansion rate and is spatiotemporally constant. The parameters in our model were normalized by $\lambda^{<0,0>}$ , $\sqrt{a_{\text{o}}}$ , and $\gamma^{<0>}$ , and their dimensionless parameters are represented with a prime, e.g., $K_{\text{a}}^{\prime <c>}=K_{\text{a}}^{<c>}\sqrt{a_{\text{o}}}/\lambda^{<0,0>}$ , $\varepsilon^{\prime }=\varepsilon \gamma^{<0>}\sqrt{a_{\text{o}}}/\lambda^{<0,0>}$ .

## 3 Results

### 3.1 Differential Area Elasticity Contributes to Cell Cluster Elongation

We previously reported that a cell cluster (i.e., type 1 cells in this study) elongates on a uniformly expanding field in the absence of surrounding cell populations (type 0 cells in this study). In real tissues, an epithelial cell cluster is not usually isolated but is instead surrounded by other cell populations. We theoretically searched for conditions under which the cell cluster can elongate even in the presence of surrounding cell populations.

**FIGURE 2:**
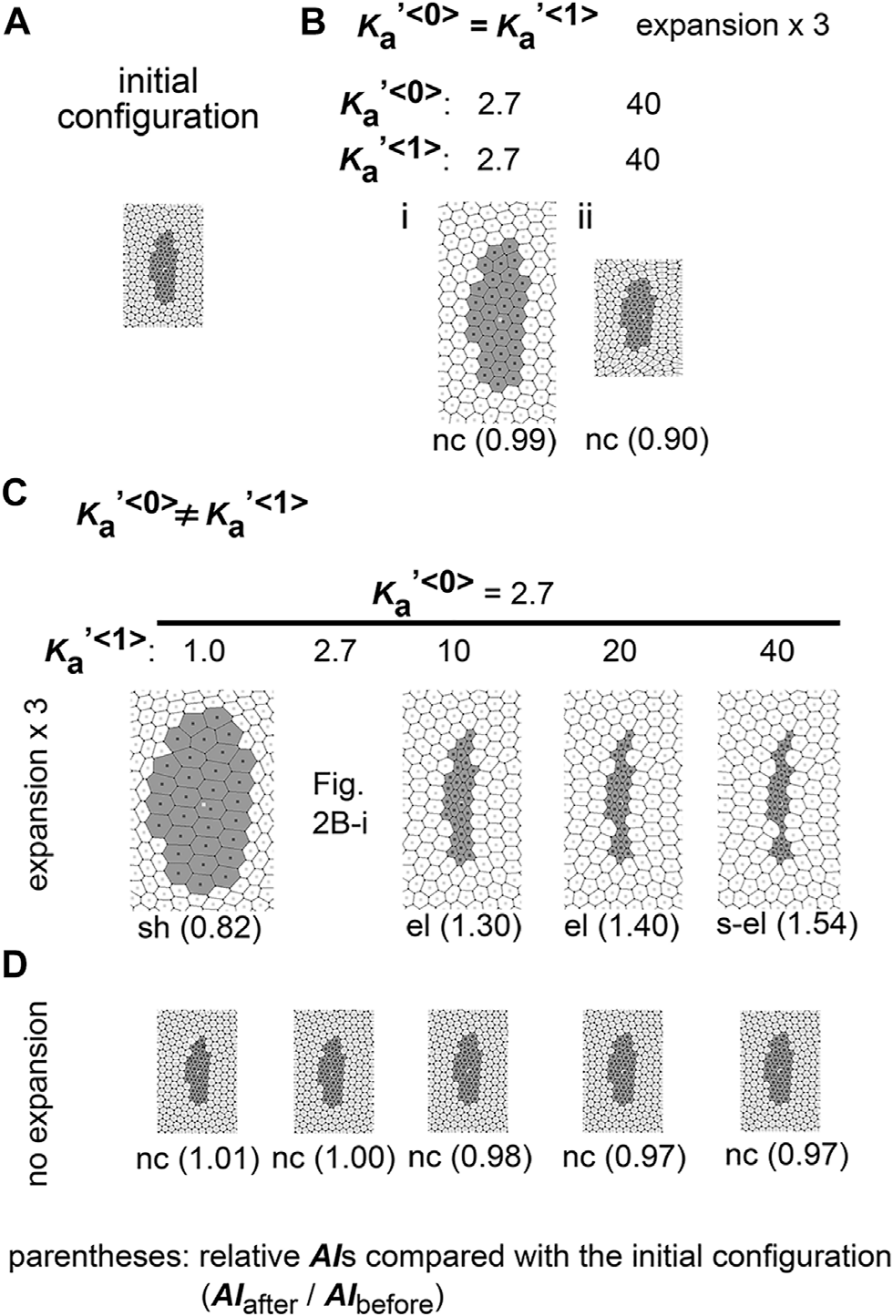
Difference in area elasticity causes elongation of cell cluster on expanding field. **(A)** The initial configuration of simulation is shown. The gray and white cells are type 1 and type 0, respectively. The whole view of the configuration is provided in Supplementary Figure S1. **(B)** Simulation outcomes under the same value between $K_{\text{a}}^{\prime <0>}$ and $K_{\text{a}}^{\prime <1>}$ are shown. $K_{\text{a}}^{\prime <0>}=K_{\text{a}}^{\prime <1>}=2.7$ in the left panel and $K_{\text{a}}^{\prime <0>}=K_{\text{a}}^{\prime <1>}=40$ in the right panel. The fields were expanded by three times in area (expansion ×3). The scales of these images are the same each other. The relative *AI*s are shown with the morphological categories [“no change (nc)”]. **(C)** Simulation outcomes under different values between $K_{\text{a}}^{\prime <0>}$ and $K_{\text{a}}^{\prime <1>}$ are shown; $K_{\text{a}}^{\prime <0>}\neq K_{\text{a}}^{\prime <1>}$ . $K_{\text{a}}^{\prime <0>}$ was fixed at 2.7. The fields were expanded by three times in area. The scales of these images are the same as those in B. The relative *AI*s are shown with the morphological categories [“elongation (el)”, “strong elongation (s-el)”, or “shrinkage (sh)”]. **(D)** Simulation outcomes under the same values of $K_{\text{a}}$ s as C are shown except that the fields were not expanded (no expansion). The scales of these images are the same as those in C. The relative *AI*s are shown with the morphological categories. Simulation results obtained using different area elasticity values are provided in Supplementary Figure S2.

First, we performed simulations using conditions under which type 1 and type 0 cells have the same parameter values, namely: $K_{\text{a}}^{<0>}=K_{\text{a}}^{<1>}$ , $\gamma^{<0>}=\gamma^{<1>}$ , and $\lambda^{<0,0>}=\lambda^{<1,1>}=\lambda^{<0,1>}$ . An initial configuration for the cells is shown in Figure 2A. We set a slightly elongated initial configuration because we previously showed that this anisotropic configuration is a prerequisite for elongation (Koyama and Fujimori, 2020). In other words, we examined whether this initially elongated state will be enhanced on the expanding field and result in more elongated states. The field was expanded under a constant expansion rate ε, and when the field size increased by three times (Figure 2B, expansion × 3), we observed the shapes of the cell clusters. Note that, due to the continuously expanding field, the cell clusters are not expected to reach steady states as we previously reported (i.e., non-equilibrium system) (Koyama and Fujimori, 2020). The cell cluster was enlarged due to friction forces from the expanding field (Figure 2B-i *vs.* Figure 2A). An index of asymmetry/elongation (*AI*) of a cell cluster was defined as described in the Materials and Methods section. If a cell cluster (type 1) is circular, *AI* becomes 1.0. If a cell cluster is elongated, *AI* becomes larger than 1.0. To evaluate the change in *AI*s before and after the simulation, we calculated the *AI* relative to that of the initial configuration (i.e., relative *AI* = *AI*_after_ */AI*_before_). The relative *AI* value was 0.99 (Figure 2B-i), indicating that the elongation of the cell cluster was not enhanced. When the values of $K_{\text{a}}$ s were set to be different from Figure 2B-i under a constraint of $K_{\text{a}}^{<0>}=K_{\text{a}}^{<1>}$ , the relative *AI* value was also ∼1.0 (Figure 2B-ii), indicating that elongation of cell clusters is insensitive to the value of $K_{\text{a}}$ .

Next, we assigned different area elasticity values between type 1 and type 0 cells: $K_{\text{a}}^{<0>}\neq K_{\text{a}}^{<1>}$ whereas $\gamma^{<0>}=\gamma^{<1>}$ and $\lambda^{<0,0>}=\lambda^{<1,1>}=\lambda^{<0,1>}$ . If the area elasticity in type 1 cells was larger than that in type 0 cells (i.e., $K_{\text{a}}^{<1>}>K_{\text{a}}^{<0>}$ ), elongation of the cell cluster was enhanced and the relative *AI*s were higher (Figure 2C, $K_{\text{a}}^{\prime <1>}\geq 10$ ). In addition, if the area elasticity in the type 1 was smaller, the relative *AI* value of the cell cluster was decreased (Figure 2C, $K_{\text{a}}^{\prime <1>}=1.0$ ). To examine whether the elongation depends on field expansion, we performed simulations without field expansion (i.e. expansion ×1) where the cells move to find a steady state under the given values of $K_{\text{a}}$ s. In Figure 2D where all simulations were performed under the same values of $K_{\text{a}}$ s as Figure 2C, the cell clusters were not elongated. In summary, under field expansion, if $K_{\text{a}}^{<0>}=K_{\text{a}}^{<1>}$ , the relative *AI* is unchanged, and if $K_{\text{a}}^{<0>}\neq K_{\text{a}}^{<1>}$ , the relative *AI* positively correlates to $K_{\text{a}}^{<1>}/K_{\text{a}}^{<0>}$ . These results indicate that, in theory, different area elasticity values contribute to tissue elongation.

We classified the morphological patterns of cell clusters by the value of the relative *AI* as follows: strong elongation (s-el; *AI*_after_ */AI*_before_ > 1.5), elongation (el; *AI*_after_ */AI*_before_ = 1.1–1.5), no change (nc; *AI*_after_ */AI*_before_ = 0.9–1.1), and shrinkage (sh; *AI*_after_ */AI*_before_ < 0.9). In Figures 2B–D, these categories are written for each simulation outcome.

### 3.2 Differential Line Tension Between Cell–Cell Interfaces do Not Cause Cell Cluster Elongation

We tested whether the differential adhesion hypothesis can reproduce the elongation of a cell cluster: $\lambda^{<0,0>}\neq \lambda^{<1,1>}\neq \lambda^{<0,1>}$ , whereas $K_{\text{a}}^{<0>}=K_{\text{a}}^{<1>}$ and $\gamma^{<0>}=\gamma^{<1>}$ . According to this hypothesis, line tensions are derived from cortical tensions and cell–cell adhesion (Steinberg, 1963; Harris, 1976): cortical tension decreases the lengths of cell–cell interfaces, and adhesion increases the lengths, and thus, the effects of these two factors on the line tensions are opposite (i.e., [line tension] = [cortical tension]—[cell–cell adhesion]) (Maître et al., 2012 and references therein). For instance, smaller values of the line tensions can result from stronger cell–cell adhesion.

**FIGURE 3:**
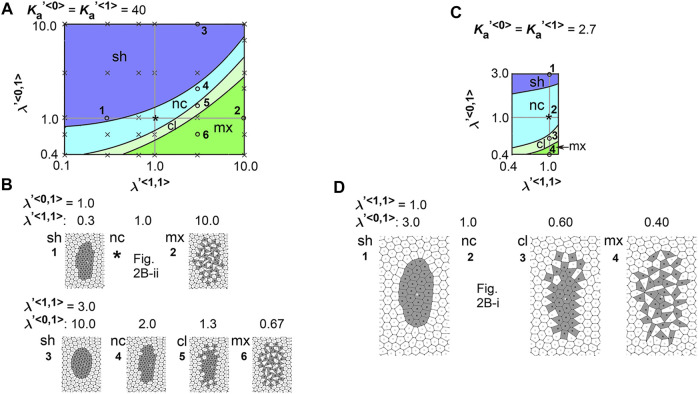
Difference in cell–cell adhesion does not cause elongation of cell cluster on expanding field. Simulations were performed using various line tension values, whereas the area elasticity and coefficient of friction were assigned the same values between the two cell types: $\lambda^{<0,0>}\neq \lambda^{<1,1>}\neq \lambda^{<0,1>}$ , whereas $K_{\text{a}}^{<0>}=K_{\text{a}}^{<1>}$ and $\gamma^{<0>}=\gamma^{<1>}$ . The fields were expanded by three times in area. Phase diagrams and examples of simulation outcomes were shown under $K_{\text{a}}^{\prime <0>}=K_{\text{a}}^{\prime <1>}=40$ **(A,B)** or under $K_{\text{a}}^{\prime <0>}=K_{\text{a}}^{\prime <1>}=2.7$ **(C,D)**. The parameter values used for each condition in B and D are plotted on the diagrams (**A,C)**, with the numbers corresponding to each condition (#1, #2, etc.). Simulation outcomes at the conditions with asterisks on the diagrams **(A,C)**, where $\lambda^{<0,0>}=\lambda^{<1,1>}=\lambda^{<0,1>}$ , were previously shown in Figure 2B-ii and Figure 2B-i, respectively. Each phase in the diagrams is colored as follow: “sh”, blue; “nc”, light blue; “cl”, light green; “mx”, green. Black crosses in A, all simulation conditions tested.

To comprehensively examine the effect of the line tensions, we performed multiple simulations under various values of the line tensions, and subsequently generated a phase diagram as follows. The parameter ranges tested in the simulations correspond to the vertical and horizontal axes in the phase diagram (Figure 3A, $\lambda^{<0,1>}$ and $\lambda^{<1,1>}$ ). For instance, a simulation condition where $\lambda^{\prime <1,1>}=\lambda^{\prime <0,1>}=1.0\left(=\lambda^{\prime <0,0>}\right)$ is marked by an asterisk, whose simulation outcome corresponds to Figure 2B-ii. From this condition, if the value of $\lambda^{<1,1>}$ was solely changed (i.e., move leftward along the x–axis), we reached a simulation condition #1, whose simulation outcome is shown in Figure 3B (#1) where the pattern was classified as shrinkage (“sh”). On the other hand, under a larger value of $\lambda^{<1,1>}$ (#2), a pattern with intermixing (“mx”) of the two cell populations was observed (Figure 3B, #2). Similarly, if the value of $\lambda^{<0,1>}$ was solely changed (i.e. move along the *y*–axis), various simulation outcomes were observed (Figures 3A, B, #3-#6). All simulation conditions are plotted on the diagram as black crosses (30–40 data points, see Materials and Methods). Then, all simulation outcomes were classified by the morphological categories defined in Figure 2. During classification, when more than two distinct cell clusters formed, the pattern was classified as either multi-cluster (cl; average cell number per cluster >1.5) or intermixed (mx; average cell number per cluster <1.5). Then, the phase diagram was divided into these morphological categories: e.g. a blue region for “sh”, a light blue region for “nc”. We could not find conditions under which cell cluster was elongated in Figure 3A.

We also performed a similar analysis under a different value of $K_{\text{a}}^{<c>}$ s and generated a phase diagram with some examples of the simulation outocomes (Figures 3C, D). A condition marked by an asterisk corresponds to Figure 2B-i where $\lambda^{\prime <0,0>}=\lambda^{\prime <1,1>}=\lambda^{\prime <0,1>}\left(=1.0\right)$ . The number of the simulation conditions used for generating the phase diagram is 30–40, but the data points are not depicted on the diagram. Similar to Figure 3A, we could not find conditions under which the cell cluster was elongated. Thus, the differential line tensions between cell–cell interfaces alone do not cause tissue elongation.

Here we interpret the above results. In the vertex models, if cell–cell adhesion is increased, the line tensions are decreased. In the phase diagrams, when $\lambda^{<1,1>}$ has a larger value (e.g., $\lambda^{\prime <1,1>}=10.0$ , and see along the x–axis), cell–cell adhesion between the type 1 and type 1 cells is decreased, and thus, the type 1 cells preferably adheres to the type 0 cells, resulting in intermixing patterns (“mx”) or generation of multiple cell clusters (“cl”). For $\lambda^{<0,1>}$ , decreased values of this parameter (e.g. $\lambda^{\prime <0,1>}=0.4$ , and see along the *y*–axis) mean increased adhesion between type 0 and type 1 cells, resulting in “mx” or “cl”, whereas increased values (e.g., $\lambda^{\prime <0,1>}=10.0$ ) prevent the cells from forming “mx” and “cl”. Moreover, such increased values in $\lambda^{<0,1>}$ decrease in the line lengths of the cell–cell interfaces between the type 0 and type 1 cells, that leads to round-up of the type 1 cell cluster emerged as the “sh” pattern.

### 3.3 Optimal Line Tension Between Cell–Cell Interfaces are Necessary in Combination With Area Elasticity to Induce Cell Cluster Elongation

**FIGURE 4:**
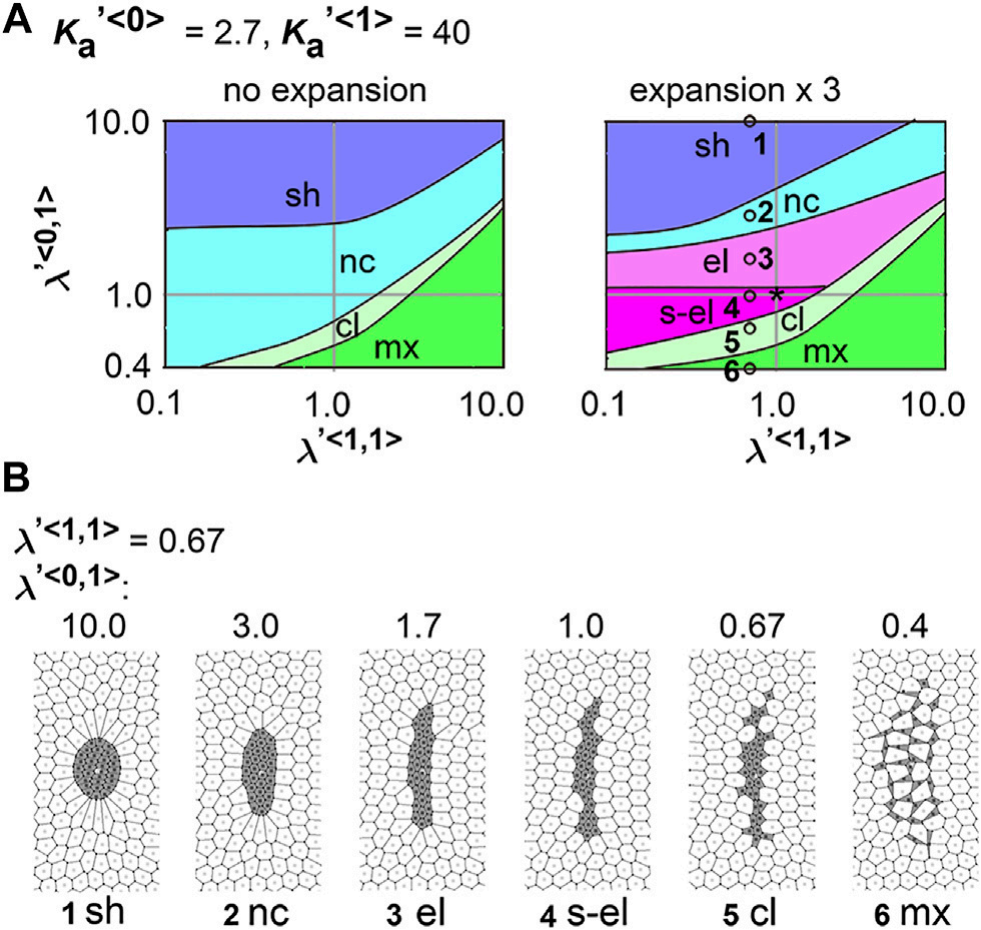
Difference in cell–cell adhesion contributes to cell cluster elongation with differential area elasticity on expanding field. Simulations were performed under conditions with various values for line tension and area elasticity, whereas the coefficient of friction was assigned the same value for both cell types: $\lambda^{<0,0>}\neq \lambda^{<1,1>}\neq \lambda^{<0,1>}$ and $K_{\text{a}}^{<0>}\neq K_{\text{a}}^{<1>}$ , whereas $\gamma^{<0>}=\gamma^{<1>}$ . Phase diagrams in the absence and presence of the uniformly expanding field are shown under $K_{\text{a}}^{\prime <0>}=2.7$ and $K_{\text{a}}^{\prime <1>}=40$ [left and right panels in **(A)**, respectively]. In the panel on the right, the fields were expanded by three times in area. Examples of simulation outcomes are shown in **(B)**, where the parameter values used for each condition in B are plotted on the diagram [**(A)**, right panel], with the numbers corresponding to each condition (#1, #2, etc.). An asterisk on the diagram [**(A)**, right panel], where $\lambda^{<0,0>}=\lambda^{<1,1>}=\lambda^{<0,1>}$ , corresponds to the condition in Figure 2C ( $K_{\text{a}}^{\prime <1>}=40$ ). Each phase in the diagrams is colored as follow: “s-el”, magenta; “el”, pink; “sh”, blue; “nc”, light blue; “cl”, light green; “mx”, green. Other simulation outcomes obtained with different values for the area elasticities are shown in Supplementary Figure S3. Simulation outcomes from a different initial configuration are shown in Supplementary Figure S4

We analyzed the combinatorial effect of line tensions and area elasticity: $\lambda^{<0,0>}\neq \lambda^{<1,1>}\neq \lambda^{<0,1>}$ and $K_{\text{a}}^{<1>}>K_{\text{a}}^{<0>}$ , whereas $\gamma^{<0>}=\gamma^{<1>}$ . Similar to Figure 3, Figure 4 shows the phase diagrams with the simulation outcomes with different line tension values for $\lambda^{<1,1>}$ and $\lambda^{<0,1>}$ . In the left panel, the field was not expanded (i.e., expansion × 1), and thus, the cells move to find a steady state under the given values of $\lambda^{<1,1>}$ and $\lambda^{<0,1>}$ . In the right panel, the field was expanded (expansion ×3). The same condition as that in Figure 2C ( $K_{\text{a}}^{\prime <1>}=40$ ) was plotted as an asterisk in Figure 3A (right panel, $\lambda^{\prime <1,1>}=\lambda^{\prime <0,1>}=1.0$ ), where the simulation outcome was classified as “strong elongation (s-el)”. In the phase diagrams, the regions of simulation conditions resulting in strong elongation (“s-el”) or elongation (“el”) are colored by magenta or pink. These regions were exclusively detected in the right panel but not the left panel, indicating that the elongation depended on field expansion. Moreover, in the right panel, if the value of $\lambda^{<0,1>}$ was solely changed (i.e., move along the *y*–axis), the cell cluster either showed no elongation (“nc” and “sh”), formation of multiple clusters (“cl”), or intermixing (“mx”) [Figure 4A, right panel, and 4B, #1, #2, #5, and #6, *vs.* #3 (“el”) and #4 (“s-el”)]. These results indicate that, for the elongation induced by the differential area elasticity, optimal line tensions were required. Other outcomes under different conditions are shown in SI (Supplementary Figures S3, S4).

### 3.4 Differential Friction Coefficient Contributes to Cell Cluster Elongation

**FIGURE 5:**
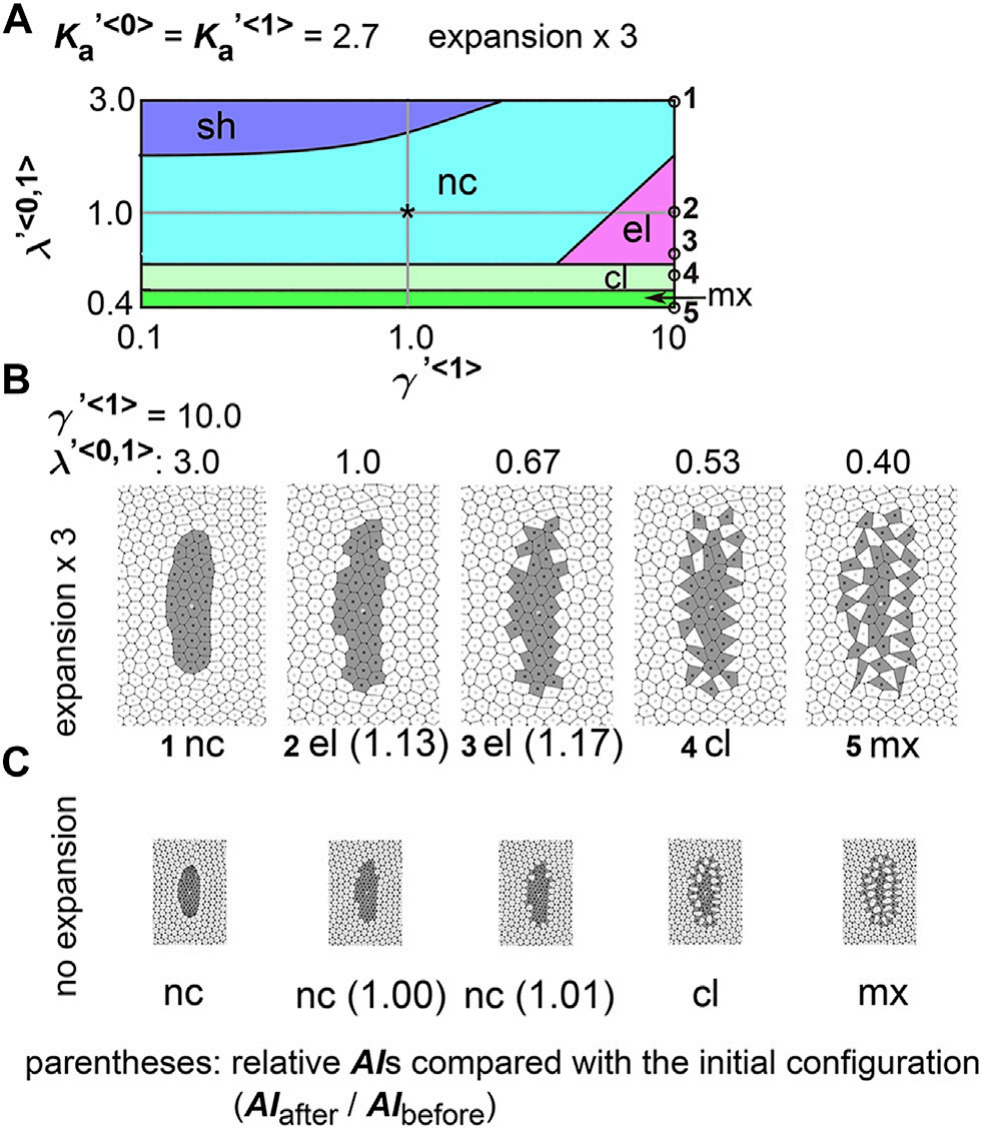
Differences in coefficient of friction cause cell cluster elongation on an expanding field. Simulations were performed under conditions with various values of the coefficient of the friction, whereas values for the area elasticity were assigned to be equal for both two cell types: $\gamma^{<0>}\neq \gamma^{<1>}$ , whereas $K_{\text{a}}^{<0>}=K_{\text{a}}^{<1>}$ . The line tensions in the type 0 and type 1 cells were made equal in value, whereas the line tension between the type 0 and type 1 cells was different: $\lambda^{<0,0>}=\lambda^{<1,1>}\neq \lambda^{<0,1>}$ . The fields were expanded by three times in area **(A,B)** or not expanded **(C)**. A phase diagram and examples of simulation outcomes were shown under $K_{\text{a}}^{\prime <0>}=K_{\text{a}}^{\prime <1>}=2.7$ . The parameter values used for each condition in B are plotted on the diagrams **(A)**, with numbers corresponding to each condition. A simulation outcome at the condition with an asterisk on the diagram **(A)** was previously shown in Figure 2B-i. Each phase in the diagrams is colored as follow: “el”, pink; “sh”, blue; “nc”, light blue; “cl”, light green; “mx”, green. The parameter values except for the field expansion were set to be the same between B and C. The scales of the images in **(B,C)** are the same each other. Other simulation outcomes obtained with different area elasticity values are shown in Supplementary Figure S5.

We determined whether the differential coefficient of the friction forces causes a cell cluster to elongate: $\gamma^{<0>}\neq \gamma^{<1>}$ , whereas $\lambda^{<0,0>}=\lambda^{<1,1>}=\lambda^{<0,1>}$ and $K_{\text{a}}^{<0>}=K_{\text{a}}^{<1>}$ . Similar to Figures 3, 4, we generated a phase diagram except that the x–axis is $\gamma^{\prime <1>}$ . The same condition as that in Figure 2B-i was plotted as an asterisk in Figure 5A where $\lambda^{\prime <0,1>}=1.0\left(=\lambda^{\prime <1,1>}=\lambda^{\prime <0,0>}\right)$ and $\gamma^{\prime <1>}=1.0\left(=\gamma^{\prime <0>}\right)$ ; the cell cluster was not elongated. From this condition, if the value of $\gamma^{<1>}$ was solely increased (i.e., move rightward along the x–axis), we reached a simulation condition #2, whose simulation outcome is shown in Figure 5B (#2) where the pattern was classified as elongation [“el (1.13)”]. Moreover, Figure 5C showed simulation outcomes in the absence of field expansion, and the cell cluster was not elongated under the same values of γ s as Figure 5B (#2). These results indicate that different values for the coefficient of friction contribute to tissue elongation, and this elongation depends on field expansion.

Next, we analyzed the combinatorial effect of line tensions and the coefficient of friction. In Figure 5A, under the same value of the coefficient of friction ( $\gamma^{\prime <1>}=10$ ), changes in the value of the line tension between the type 1 and type 0 cells (i.e., $\lambda^{<0,1>}$ ) resulted in patterns other than elongation (“el”), such as no elongation (“nc”), multiple clusters (“cl”), and intermixing (“mx”) (Figures 5A, B, #1 [“nc”], #4 [“cl”], and #5 [“mx”] *vs.* #2 and #3 [“el”]). Thus, optimal line tensions were required for the elongation induced by varying coefficients of friction force. Moreover, in the absence of field expansion, elongation of the cell clusters was not induced, whereas the other patterns were observed (Figure 5C). Other outcomes under different conditions are shown in SI (Supplementary Figure S5).

### 3.5 Cell Behavior in Mouse Notochord Elongation

In real tissues, elongation of various tissues is usually explained by directionally active cell movement that results in convergent extension (Keller et al., 2000; Honda et al., 2008) where expanding fields are not considered. By contrast, we have shown experimentally that elongation of the mouse notochord depends on an increase in volume of the amniotic cavity (Imuta et al., 2014). From our theoretical analyses in Figures 2, 5, we raised two hypotheses for field expansion–dependent tissue elongation: area elasticity–based one, and friction coefficient–based one. We determined whether the elongation of the mouse notochord is consistent with differences in area elasticity or differences in friction coefficient. According to our previous data in Figure 2C and Figure 5B-#2, the cell area in the cell cluster of interest appears to either be almost unchanged according to the area elasticity–based hypothesis or increased according to the friction–based hypothesis: the mean cell area is 1.4-fold of that in initial configuration under the area elasticity–based hypothesis, and is 5.2-fold under the friction–based hypothesis. Conversely, the cell area in the surrounding cell populations seems to increase according to both hypotheses: the mean cell area is 4.3-fold of that in initial configuration under the area elasticity–based hypothesis, and is 4.6-fold under the friction–based hypothesis.

**FIGURE 6:**
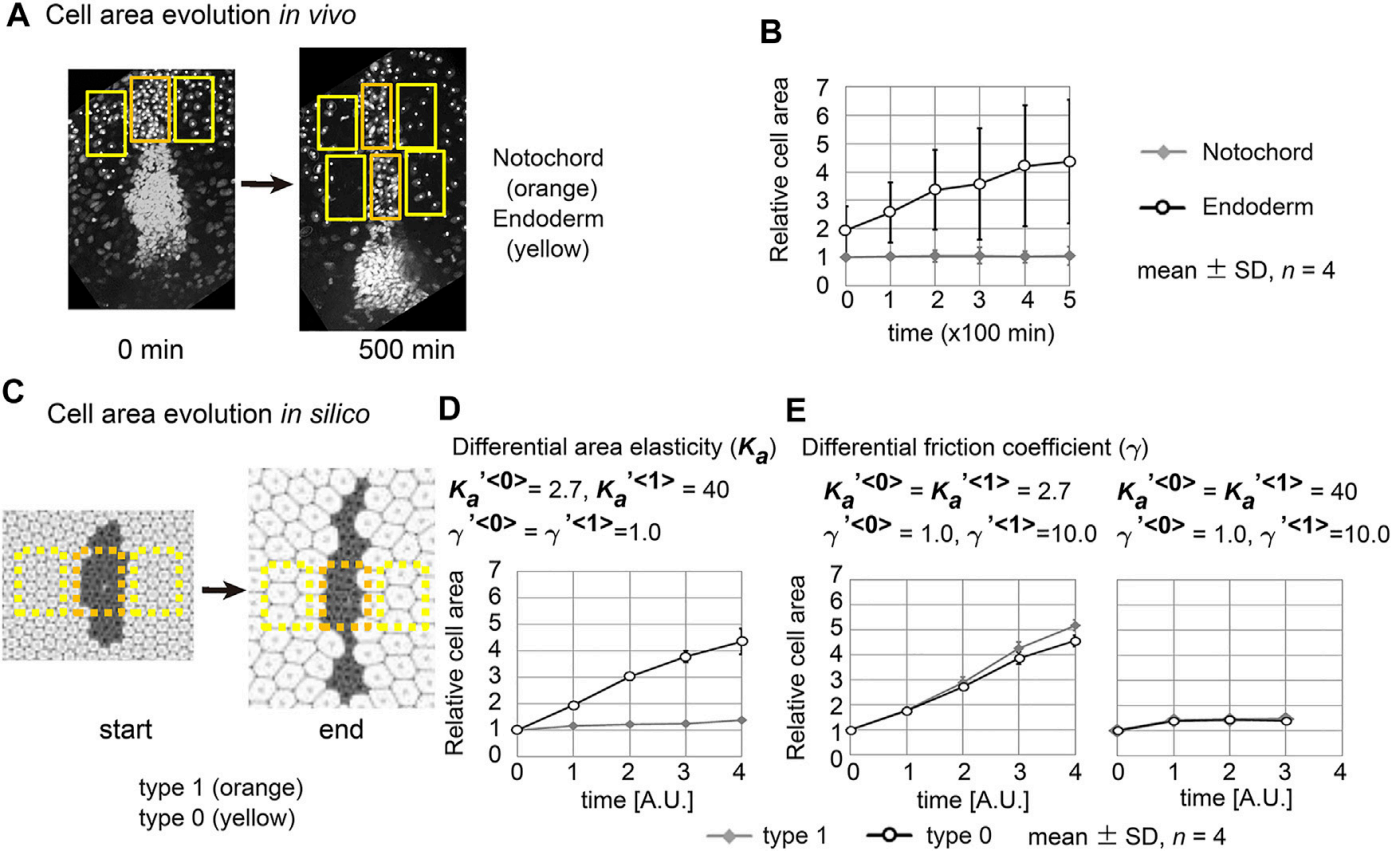
Cell behaviors in mouse notochord and their comparison with two theories. **(A)** Confocal microscopic images at two time points in the mouse notochord and the surrounding endoderm are shown. Nuclei of the cells were visualized by histone 2B fused to EGFP. The orange and yellow rectangles are regions used for measuring cell areas within the notochord and endoderm, respectively. These microscopic images were obtained from our previous work (Imuta et al., 2014). **(B)** The cell areas in the notochord and endoderm are shown with their temporal evolutions. The mean cell area in the notochord at 0 min was defined as 1.0. Four embryos were analyzed (*n* = 4). **(C)** Cell areas in simulations were measured. Simulation outcomes are indicated with orange and yellow rectangles that were used to measure cell areas in a similar manner to B. **(D)** Cell areas obtained with differential area elasticities, which were derived from the simulation outcomes in Figure 2C $\left(K_{\text{a}}^{\prime <1>}=40\right)$ , are shown. Four different initial configurations of the simulations (*n* = 4) were applied. Parameter values for area elasticities and coefficients of friction are also indicated. **(E)** Cell areas obtained with differential friction coefficients, which were derived from the simulation outcomes in Figure 5; Supplementary Figure S5, are shown in a similar manner to **(D)**. The values from type 1 and type 0 cells are nearly identical in the right panel.

We went on to measure the dynamics of the cell area both *in vivo* and in silico. *In vivo* cell areas were estimated from the densities of the cells in the notochord or the endoderm (Figure 6A and Materials and Methods). The apical cell area within the notochord was temporally unchanged, whereas that in the endoderm increased (Figure 6B). In the case of simulation data, we also estimated cell areas from the densities of the cells (Figure 6C). Under the area elasticity–based hypothesis, the cell area in the type 1 cell cluster was temporally unchanged, whereas that in the surrounding type 0 cell populations was increased (Figure 6D). Under the friction coefficient–based hypothesis, the cell areas in both the cell cluster and the surrounding cell populations were temporally increased (Figure 6E, a left panel, $K_{\text{a}}^{\prime <0>}=K_{\text{a}}^{\prime <1>}=2.7$ ). In addition, if the area elasticities in both cell types were made larger under different friction coefficients, the increases in the cell areas were restricted for both cell types but the dynamic was equivalent between the two cell types (Figure 6E, a right panel, $K_{\text{a}}^{\prime <0>}=K_{\text{a}}^{\prime <1>}=40$ ). Thus, the dynamics of the cell areas according to the area elasticity–based hypothesis are consistent with the *in vivo* dynamics but not for the friction–based hypothesis.

### 3.6 Differential Preferred Cell Area Contributes to Tissue Elongation in Field Expansion–independent Manner

Differentiation of cells may contribute to the differences in area elasticity as examined in the previous section. On the other hand, cell differentiation may directly change the preferred cell area $a_{\text{O}}$ defined in Eq. 1. As shown above, the cell areas were specifically increased in the surrounding cell populations (i.e., endodermal cell/type 0 cells). Such specific increase in the cell areas would be reproduced by increases in the preferred cell area. As an alternative to the differential area elasticity–based hypothesis, we investigated the influence of differential preferred cell area on not only the specific increase in the cell areas but also tissue elongation.

**FIGURE 7:**
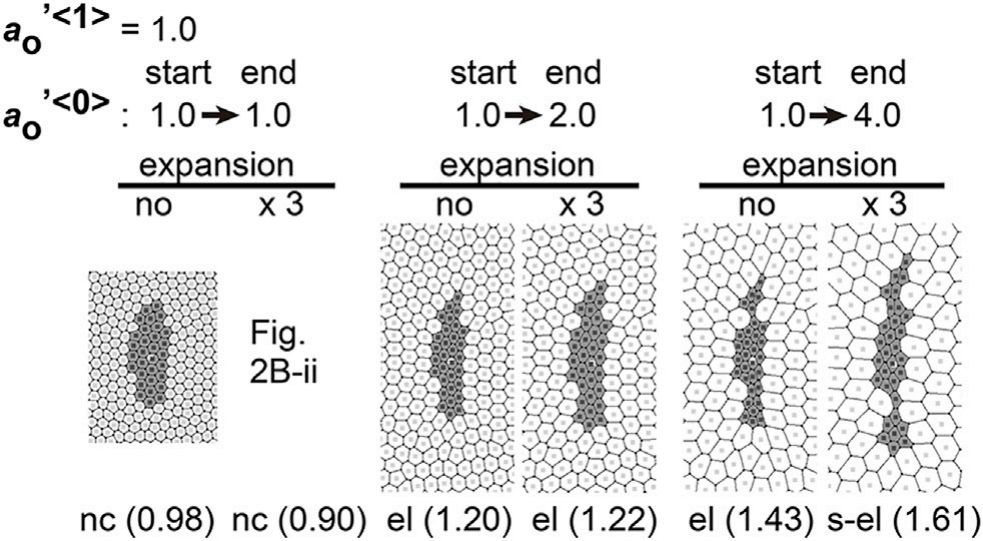
Differences in preferred cell areas cause cell cluster elongation in a manner independent to field expansion. Simulations were performed under conditions with temporal changes in the preferred cell areas in the type 0 cells, whereas the values of other parameters were assigned to be equal for both two cell types: $a_{\text{O}}^{\prime <0>}=$ temporal increase, whereas $a_{\text{O}}^{\prime <1>}=const.\left(=1.0\right)$ , $\lambda^{\prime <0,0>}=\lambda^{\prime <1,1>}=\lambda^{\prime <0,1>}\left(=1.0\right)$ , $K_{\text{a}}^{\prime <0>}=K_{\text{a}}^{\prime <1>}\left(=40\right)$ , and $\gamma^{\prime <0>}=\gamma^{\prime <1>}\left(=1.0\right)$ . The values of $a_{\text{O}}^{\prime <0>}$ at the start and end of the simulations are shown (e.g., start [1.0] → end [4.0]). Two scenarios of the temporal changes were applied (end [2.0] and [4.0]), in addition to a scenario of temporally no change (end [1.0]). In the two scenarios, the values are linearly increased during the simulations [i.e., $a_{\text{O}}^{\prime <0>}\left(t\right)=ct+a_{\text{O}}^{\prime <1>}\left(0\right)$ , where *t* is time, and *c* is const.]. The categories of the simulation outcomes are described with the relative *AI* values (parentheses).

We assumed specific increases in the preferred cell areas in the type 0 cells where this parameter was set to temporally increase during simulations. In Figure 7, $a_{\text{O}}^{\prime <1>}$ and $a_{\text{O}}^{\prime <1>}$ denote $a_{\text{O}}^{\prime }$ in the type 1 and type 0 cells, respectively. The value of $a_{\text{O}}^{\prime <1>}$ was fixed at 1.0. In addition, the parameter normalization described in *A Uniformly Expanding Field* was performed using $\sqrt{a_{\text{o}}^{<1>}}$ . The value of $a_{\text{O}}^{\prime <0>}$ was temporally changed from 1.0 at the start of simulations to 1.0, 2.0, or 4.0 at the end of simulations (Figure 7, start and end). The cell areas in the type 0 cells were specifically increased in the right panels (end = 4.0), regardless to the field expansion (expansion = “no” and “×3”). Furthermore, the type 1 cell cluster was elongated under the field expansion (expansion = “×3”). However, elongation of the type 1 cell cluster was also observed even without the field expansion (expansion = “no”). These results indicate that differential preferred cell areas cause tissue elongation, but the elongation does not depend on field expansion. Because the elongation of the mouse notochord depends on the field expansion (Imuta et al., 2014), this differential preferred cell area hypothesis is not consistent with the *in vivo* situations.

### 3.7 Experimental Measurement of Cellular Stiffness in Mouse Notochord Elongation

**FIGURE 8:**
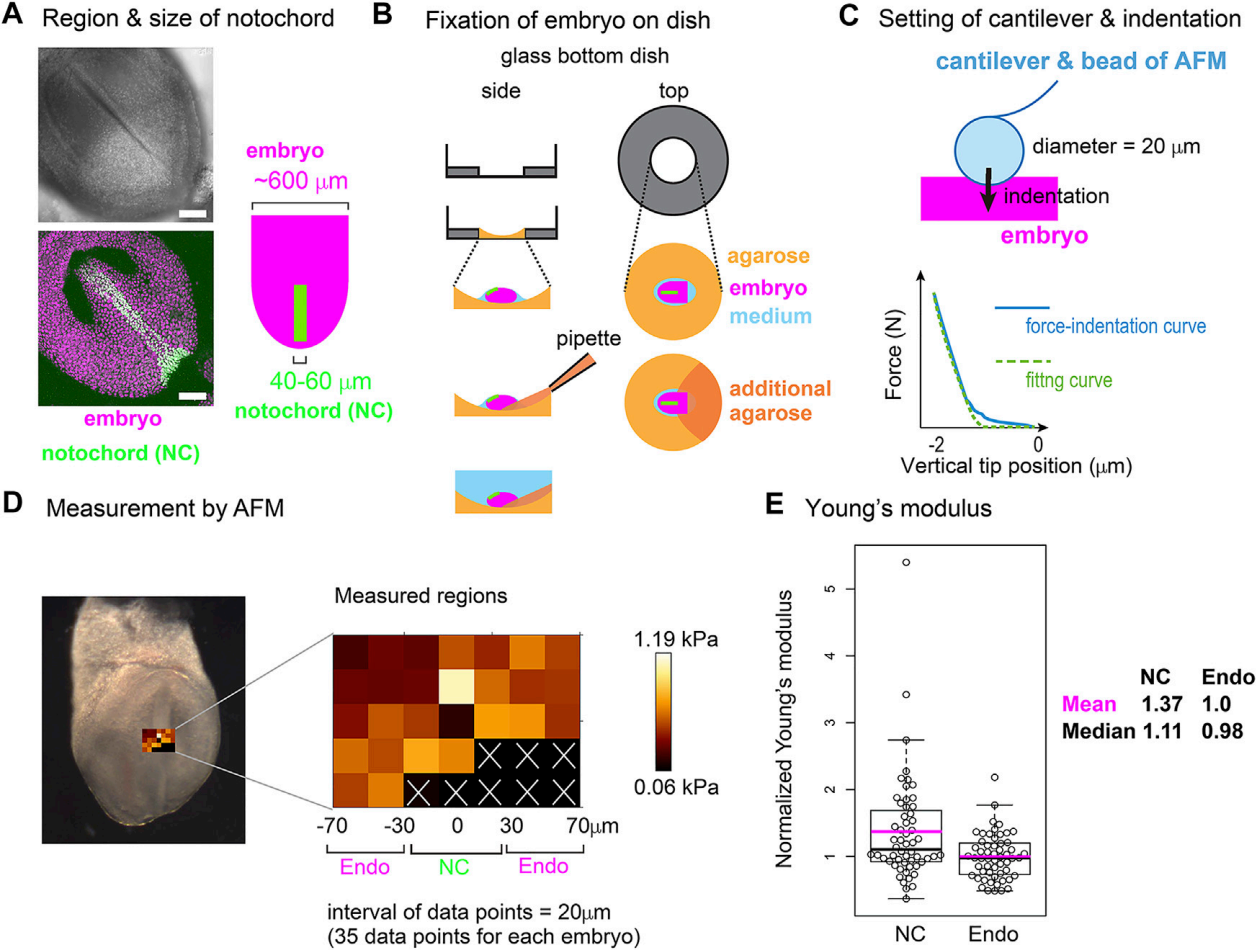
Young’s modulus measured using AFM in mouse notochord and endoderm. **(A)** Microscopic images of a mouse embryo are shown. Nuclei of the notochord cells were labeled with nuclear-EGFP (green), and nuclei of all embryonic cells including the endodermal cells as well as the notochord cells were labeled by histone 2B-mCherry (magenta). Upper panel, brightfield; bottom panel, a maximum intensity projection image constructed from confocal fluorescent images; left panel, illustration of an embryo and the notochord. Typical widths of the notochord and embryo are written. **(B)** Preparation procedure of embryo for AFM is illustrated. An embryo (magenta) is placed on an agarose gel (light orange) in a glass-bottom dish, and a part of the embryo is overlaid by an additional agarose gel (dark orange) before medium (blue) is added. Side and top views are shown. **(C)** AFM cantilever assembly and indentation. A bead of 20 μm diameter was attached to the cantilever as described in the Materials and Methods section. A force–indentation curve is schematically illustrated with a fitting curve which is used for calculating the Young’s modulus. The x–axis is the depth of indentation. Examples of real force–indentation curves are shown in Supplementary Figure S6. **(D)** A spatial map of Young’s modulus measured by AFM is shown. In the left panel, a brightfield microscopic image is provided where the regions subjected to the AFM measurement are also shown. In the middle panel, the Young’s modulus for each region in the embryo is shown with a 20 μm spatial interval. In regions marked by white crosses, AFM measurements failed to be carried out. The regions of the notochord and endoderm were estimated by the width of the notochord. NC, notochord; Endo, endoderm. In the right panel, a color bar of Young’s modulus is shown. **(E)** Young’s moduli in the notochord and the endodermal regions are compared. The mean value in the endodermal regions was set at 1.0, and the relative values were plotted. Four embryos with several tens of data points were analyzed with total data points = 56 in both NC and Endo. Boxplots are shown where the boxes extend from the lower to upper quartiles and the whiskers indicate the 1.5–interquartile ranges. The *p*-value calculated using the Mann–Whitney–Wilcoxon test was 0.006. Magenta bars, mean; black bars, median; NC, notochord; Endo, endoderm. According to this boxplot, there are two or one outlier(s) located outside of the whiskers for NC and Endo. When these outliers are excluded, the statistic values become as follows: mean = 1.25 (NC), 0.98 (Endo), and median = 1.07 (NC), 0.98 (Endo), and *p*-value = 0.008. The mean values of the four individual embryos (#1–#4) are as follows (notochord, endoderm), #1 (0.37 [kPa], 0.22), #2 (0.48, 0.36), #3 (0.55, 0.47), and #4 (0.63, 0.53).

To further validate the area elasticity–based hypothesis in the mouse notochord, we measured cellular stiffness. To the best of our knowledge, no method for measuring area elasticity directly has been established to date. We used atomic force microscopy (AFM) that has been used to measure cellular stiffness (Young’s modulus) (Addae-Mensah and Wikswo, 2008; Barriga et al., 2018; Kinoshita et al., 2020). The difference between area elasticity and the Young’s modulus is discussed in the Discussion section. A mouse embryo is shown in Figure 8A where the nuclei in the notochord cells were labeled by green fluorescent proteins (GFP) and all the nuclei including the endodermal cells were labeled by other fluorescent proteins (mCherry) (Figure 8A). A mouse embryo was placed on an agarose gel (Figure 8B, light orange), and subsequently, a part of the embryo was overlaid by an additional agarose gel (Figure 8B, dark orange). The Young’s modulus of the regions of the notochord or the surrounding endoderm was measured through indentation of the cantilever with a bead of diameter = 20 μm attached and subsequent acquisitions of force–indentation curves (Figure 8C; Supplementary Figure S6). A spatial map of the Young’s modulus around the notochord and endoderm was obtained with interval = 20 μm (Figure 8D, “Measured regions”), where the colors for each data point reflect the values of the Young’s modulus as defined by the color bar. In this embryonic stage, the widths of the notochord were 40–60 μm (Figure 8A) as described in Materials and Methods *Atomic Force Microscopy*. Therefore, the three columns adjacent to the midline were expected to be on the notochord (Figure 8D, NC) and the outer two columns for each side were on the endoderm (Figure 8D, Endo). Data points that did not yield a clear force–indentation curve were omitted from the data analysis (Figure 8D, white crosses). The mean values of the Young’s moduli in four embryos were calculated (Materials and Method *Atomic Force Microscopy*), and the values are 0.51 [kPa] in the notochords and 0.40 [kPa] in the endoderms. Figure 8E is the comparison of the Young’s moduli between the notochord and the endoderm, where the Young’s moduli were normalized by the mean value in the endoderms. The Young’s modulus of the notochord regions was larger than that of the endodermal regions. These results suggest that the Young’s modulus of the notochord is higher than that of the endoderm. In addition, stiffnesses of various cells and tissues are 0.01–10 [kPa], and our measurement results are within this range (Davidson et al., 1999; Zhou et al., 2009).

## 4 Discussion

In this study, we theoretically investigated the potential roles of area elasticities and coefficients of friction in tissue elongation on a uniformly expanding field. In the case that the area elasticities or coefficients of friction differed between the cell cluster and the surrounding cell populations, the cell cluster is elongated as summarized in Table 1. By contrast, differences in cell–cell adhesion based on the differential adhesion hypothesis cannot cause the cell cluster to elongate. On the other hand, the differences in the preferred cell areas lead to elongation even without field expansion. The two hypotheses based on the area elasticity and the friction coefficient lead to different cellular behaviors; the apical cell areas in the surrounding cell populations are increased in both hypotheses, whereas the areas in the cell cluster of interest are either almost unchanged according to the former hypothesis or increased according to the latter (Table 1). Therefore, the elongation of the mouse notochord may be explained by the area elasticity–based hypothesis, though we do not exclude the possibility that these hypotheses simultaneously contribute to the elongation.

**TABLE 1:** Summary of comparison of phenomena; *in vivo vs.* different models under uniformly expanding field.

Phenomena	*In vivo*	Uniformly expanding field			
	*Mouse notochord*	*Hypothesis A*	*Hypothesis B*	*Hypothesis C*	*Hypothesis D*
		Differential cellular stiffness	Differential friction coefficient	Differential adhesion/tension	Differential preferred cell area
Elongation of cell cluster/tissue	+^a^	+	+	−	+
Relative increase in cell area (NC *vs.* END)	NC < END	NC < END	NC = END	Not determined	NC < END
Dependency of elongation on field expansion	Yes^a^	Yes	Yes	No elongation under field expansion	No

NC, notochord; END, endoderm.

a Imuta et al. (2014).

The elements consistent to the *in vivo* situations are shaded by gray.

Measurement of Young’s modulus through AFM suggests that the notochord is stiffer than the endoderm. The Young’s modulus of the notochord and endoderm differed by ∼1.4 times, whereas the difference in the area elasticity in our simulations was up to 10 times. The Young’s modulus differs from the area elasticity, although both are measures of cellular stiffness. During the AFM–based measurement, the direction of indentation is parallel to the apico–basal axis. On the other hand, the area elasticity is related to the change in apical cell area whose direction is perpendicular to the apico–basal axis. Nevertheless, the change in apical cell area and in the apico–basal height should be related under conserved cell volume; the increase of apical cell area should lead to the decrease in the apico–basal height, and vice versa. Although we do not have quantitative relationship between the Young’s modulus and the area elasticity, we suppose that the Young’s modulus reflects, at least partially, the area elasticity. In general, cell stiffness can differ by over an order of magnitude (Zhou et al., 2009; Swift et al., 2013). The notochord in chordates is believed to provide stiffness of their bodies (Hejnol and Lowe, 2014; Corallo et al., 2015), and the notochord in *Xenopus laevis* was experimentally shown to be several to several tens times stiffer than the endoderm (Zhou et al., 2009). In contrast to the Young’s modulus and the area elasticity, both of which reflect mechanical properties of cells or tissues, the preferred cell area is not a parameter reflecting mechanical properties. Therefore, the Young’s modulus is not related to the preferred cell area, and the differences in the Young’s moduli between the two cell populations supports the differential area elasticity–based hypothesis.

The mechanism of tissue elongation on a uniformly expanding field was mathematically/analytically analyzed in our previous work where an isolated cell cluster was solely considered (i.e., type 1 cell) (Koyama and Fujimori, 2020). If a cell cluster is enlarged by external forces (i.e., $a_{n}$ becomes $>>a_{\text{O}}$ ) and then released from the forces in the absence of field expansion, the cell cluster decreases its area, and eventually reaches a natural state where each cell area becomes nearly $a_{\text{O}}$ . Interestingly, during this process, the cell cluster transiently enhances its anisotropy, leading to the emergence of an elongated shape. Note that this anisotropy is finally diminished when the cell cluster reaches the steady state. On an expanding field, this enhancement of anisotropy is continued because the cell cluster is maintained under an enlarged state (i.e., $a_{n}>>a_{\text{O}}$ ), and thus, the cell cluster is continuously elongated. Furthermore, our analytical approach showed that this enhancement of anisotropy depends on both area elasticity and increased cell area as shown in the eq. S16 of our previous work (Koyama and Fujimori, 2020).

On the other hand, our present study showed that differential area elasticity is effective for tissue elongation in the presence of surrounding cell populations. This seems to be consistent with our above analytical conclusion. Intuitively, if area elasticity in the surrounding cell populations (type 0) is significantly smaller than that in the cell cluster of interest (type 1), forces that the type 1 cells receive from the type 0 cells become negligible, leading to a situation equivalent to an isolated cell cluster. Imagine that, if a very stiff material is surrounded by very soft materials, the soft materials can have negligible influences to the behavior of the stiff material. We also showed that friction coefficient is effective for tissue elongation. According to Eq. 4, the apparent friction forces that a vertex receives from the expanding field is $F_{\text{e|}h}=\gamma_{\text{V}}V_{\text{e}}$ , meaning that the force values are increased under larger friction coefficient $\gamma_{\text{V}}$ . The apparent friction forces are effective for increasing cell areas; the increase results in the enhancement of anisotropy according to our previous analytical conclusion. In consistent with this, under the differential friction coefficients, the cell areas in type 1 become slightly larger than those in type 0 in our simulation (Figure 6E).

Mechanisms of tissue elongation have been experimentally and theoretically studied well (Keller et al., 2000; Honda et al., 2008). In these mechanisms, a cell cluster of interest is assumed to have an intrinsic activity of directional cell movement or anisotropic property of cell–cell interfaces (Zajac et al., 2000; Zajac et al., 2003; Honda et al., 2008), whereas any expanding field is not considered. Our present study shed light on a possible contribution of an expanding field to pattern formation, and consequently, the involvement of the area elasticity and the coefficient of the friction in tissue elongation was demonstrated. Expansion of a cavity and its role in morphogenesis has been discussed in both mouse and fish (Trinkaus, 1984; Tam and Behringer, 1997). Friction between fields and cells should exist in development of various multicellular systems including germ layers (Butler et al., 2009; Reig et al., 2017; Smutny et al., 2017), epidermis during pregnancy (Ichijo et al., 2017), and cells in contact with other cell layers such as smooth muscle layers or with external structures such as eggshells (Shyer et al., 2013; Koyama et al., 2016; Münster et al., 2019). Cell–extracellular matrix interaction is important for morphogenesis (Ryan et al., 2001; Goodwin et al., 2016) and would also be related to the friction forces. Further investigation is required to clarify what kind of real tissues our two hypotheses apply to.

## 5 Materials and Methods

### 5.1 Mathematical Model and Analysis

The implementation of our mathematical model is essentially the same as that in our previous article. The surrounding cell populations have an outer boundary as shown in Supplementary Figure S1, and cropped views are shown in Figures 2–5, 7. Total simulation time $\left(t^{\prime }=t\lambda^{<0,0>}/\left(\gamma^{<0>}\sqrt{a_{\text{o}}}\right)\right)$ was fixed at 30 as a dimensionless time. Within this time period, ε was set so that the sizes of expanding fields become three times of the initial situations [i.e., exp (*εt*) = 3]. For the generation of all the phase diagrams, 30–40 simulation conditions were applied with some additional conditions for fine resolutions in specific regions as exemplified in Figure 3A. Simulations were performed using the Euler method.

The definition and measurement of the asymmetry/elongation index (*AI*) were reported previously (Koyama and Fujimori, 2020). The length of the longest axis of a cell cluster was measured as the maximum caliper. *AI* was defined as *AI = Feret*/*D*_circle_, where *Feret* is the maximum caliper, and *D*_circle_ is the diameter of a circle with the same area as the cell cluster. Thus, *AI* is 1.0 in a circle and is increased in an elongated shape. Simulation outcomes were converted to TIFF images, and the *Feret* and the area of a cell cluster were measured using ImageJ (*Feret* is prepared as a measurement option for ImageJ. https://imagej.nih.gov/ij/docs/menus/analyze.html#set).

The definition of the patterns in Figures 2–5, 7 is described in the main text. Briefly, when all type 1 cells formed a single cluster, the pattern was categorized as either “s-el”, “el”, “nc”, or “sh”, according to the ratio of *AI* after the simulation to *AI* before the simulation (*AI*_after_/*AI*_before_); when more than two separate clusters formed, the pattern was categorized into either “cl” or “mx”.

### 5.2 Image Analysis for Estimating Cell Area

Cell areas in the mouse notochord and endoderm were estimated as follows. A rectangular region was defined on the notochord at 0 min (Figure 6A, left panel, 0 min, orange rectangle). Rectangular regions with the same width as the above were defined on the endodermal regions, which were also adjacent to the rectangle on the notochord (Figure 6A, 0 min, yellow rectangles). For images after time evolution (Figure 6A, 500 min), rectangular regions were defined on the notochord, whose widths may differ from that at 0 min. Rectangular regions set on the endodermal regions at 500 min have the same widths as at 0 min. Cell areas in these regions were defined as [the area of the rectangle/the nuclear numbers]. Similar procedures were also carried out for simulation outcomes as shown in Figure 6C with orange and yellow rectangles.

### 5.3 Mouse Embryo

The notochord cells were identified by the expression of *Brachyury* (*T*). The *Brachyury*-expressing cells were labeled by nuclear enhanced green fluorescent protein (EGFP) as reported previously (Imuta et al., 2013) (Acc. No. CDB0604K; http://www2.clst.riken.jp/arg/mutant%20mice%20list.html). Briefly, knock-in mice expressing both Brachyury and nuclear EGFP from the endogenous *Brachyury* gene locus were used. All embryonic cells including the endodermal cells were labeled with mCherry-fused H2B (histone 2B proteins) expressed under the control of a ubiquitous promoter, *ROSA26*, as we reported previously (Abe et al., 2011). By mating these two mouse lines following a subsequent generation, we eventually obtained a mouse line that is both homozygous for *H2B-mCherry* and heterozygous for *Brachyury* with the nuclear *EGFP* gene. By mating males from this mouse line with ICR female mice (Japan SLC), embryos expressing both H2B-mCherry and nuclear EGFP were obtained with 50% probability.

### 5.4 Atomic Force Microscopy

Embryos described above were isolated on embryonic day 7.5. The embryos were placed in DMEM containing HEPES with 50% FBS on ice. The concentration of the agarose (BMA, SeaKem GTG, cat. 50,070) in Figure 8B is 1% melted in PBS. The embryos were placed on the agarose (Figure 8B, light orange), and the medium was almost removed. Finally, a small amount of 1% agarose was added to anchor the embryos (Figure 8B, dark orange). DMEM containing HEPES with 50% FBS was added on ice again. Thirty minutes before the AFM measurement, the above embryos were transferred to an incubator at 37°C, and the DMEM medium was replaced with PBS just before the AFM measurement. Four distinct embryos were subjected to AFM measurement, and for each embryo, several tens of measurement points were defined as described in Figure 8D. For each embryo, mean values of both the notochord and the endoderm were calculated, and the mean values of the four embryos are shown in the legend of Figure 8E. Calculation of the normalized Young’s moduli in Figure 8E was performed for each embryo: all data points in one embryo were normalized by the mean value of the embryo’s endoderm.

We could not identify the exact location of the notochord during AFM, because our AFM has a bright field microscope but not a good fluorescent one. Alternatively, we independently performed a fluorescent imaging using a confocal fluorescent microscope (Nikon A1, Japan) as shown in Figure 8A, and estimated the width of the notochord at 40–60 μm. Because the midline of the notochord was distinguishable in the bright field microscopy of the AFM (Figure 8D), we assumed that the notochords were located around the midline and 40–60 μm width.

AFM measurements were conducted as previously described (Kinoshita et al., 2020). In brief, a JPK Cellhesion 200 (Bruker) fitted with an x/y-motorized stage and mounted on a macro zoom microscope (Axio Zoom.V16, Zeiss) was used. Customized AFM probes (Novascan) were prepared by attaching borosilicate beads (Figure 6C, 20 μm diameter) to tipless rectangular silicon cantilevers (350 μm long, 32.5 μm wide, 1 μm thick; nominal spring constant 0.03 N/m, MikroMasch). Force–indentation curves (maximum indentation force: 3 nN, approach speed: 5 μm/s) were acquired every 20 μm apart in a bidirectional raster scan (Figure 8D), leading to that data points on the three columns adjacent to the midline were expected to be on the notochord. Cell elasticity (Young’s modulus) values on the tissue surface were calculated based on the Hertz model and mapped onto brightfield images using the JPK data processing software (Bruker).

## Data Availability

The raw data supporting the conclusion of this article will be made available by the authors, without undue reservation.
