# Supplementary material for: Differential Cellular Stiffness Contributes to Tissue Elongation on an Expanding Surface
Source: Front Cell Dev Biol. 2022 Mar 29;10:864135. doi: 10.3389/fcell.2022.864135 (PMC9001851; doi:10.3389/fcell.2022.864135)
Supplement: Supplementary file 1 [file DataSheet1.PDF]

Supplementary Material

1 Supplementary Figures and Tables

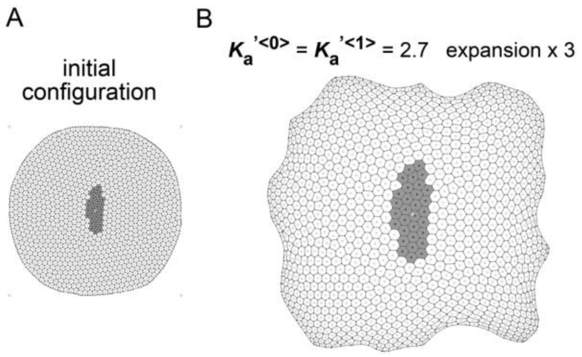

Supplementary Figure 1 (related to Fig. 2): Overview of simulation.
A. Overview of the initial configuration of simulations in Figs. 2–5, 7.
B. Overview of the simulation outcome in Fig. 2B-i.

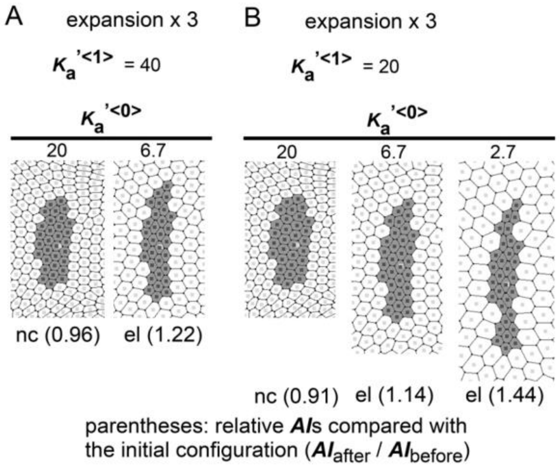

Supplementary Figure S2 (related to Fig. 2): Simulation outcomes given various area elasticity values.

A. Simulation outcomes given different area elasticity values for type 0 cells ($K_a'^{<0>}$) (20 and 6.7). The area elasticity in the type 1 cells ($K_a'^{<1>}$) was set at 40. The fields were expanded by three times in area. The relative *AI* are shown at the bottom of each panel in a similar manner to Fig. 2. These images are all of the same scale.

B. Simulation outcomes under different values of $K_a'^{<0>}$ are shown (20, 6.7, and 2.7). $K_a'^{<1>}$ was set at 20. The fields were expanded by three times in area. The relative *AI* are shown in a similar manner to A. These images are all of the same scale.

In both A and B, the cell cluster was only elongated under conditions with $K_a^{<1>} > K_a^{<0>}$ but not with $K_a^{<1>} < K_a^{<0>}$.

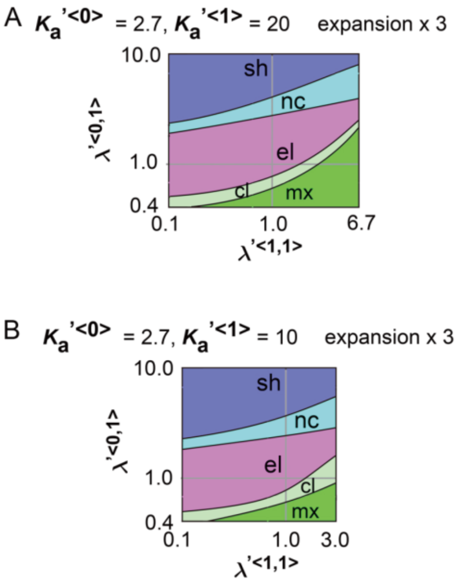

Supplementary Figure S3 (related to Figure 4): Effect of differences in cell–cell adhesion on elongation of cell cluster given various values for area elasticities.

Simulations were performed and phase diagrams were generated in a similar manner to Fig. 4, except for different combinations of the values of the area elasticities in the type 1 and 0 cells. In A, $K_a'^{<1>}$ was set at 20, whereas, in B, $K_a'^{<1>}$ was set at 10. These phase diagrams showed an almost similar pattern to Fig. 4A.

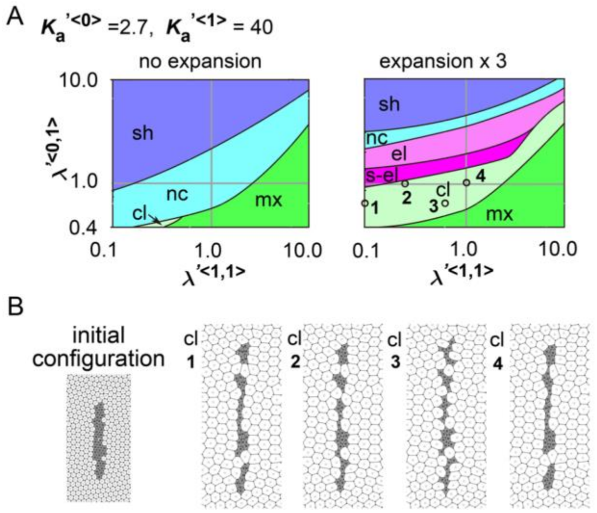

Supplementary Figure S4 (related to Fig. 4): Difference in cell–cell adhesion contributes to pattern formation of cell cluster with differential area elasticity on an expanding field.

Simulations were performed and phase diagrams were generated in a similar manner to Fig. 4, except for a different initial configuration of simulations (B, initial configuration), which forms a more elongated shape compared with that in Fig. 2A. The phase diagrams (A) show a nearly identical pattern to Fig. 4A. In addition, as shown in B, a single cluster of cells sometimes formed a linear array of multiple distinct cell clusters, and such a pattern is observed in various developing tissues (Meilhac et al., 2003; Tzouanacou et al., 2009).

Meilhac, S. M., Kelly, R. G., Rocancourt, D., Eloy-trinquet, S., Nicolas, J., and Buckingham, M. E. (2003). A retrospective clonal analysis of the myocardium reveals two phases of clonal growth in the developing mouse heart. *Development* 130, 3877–3889. doi:10.1242/dev.00580.

Tzouanacou, E., Wegener, A., Wymeersch, F. J., Wilson, V., and Nicolas, J.-F. (2009). Redefining the Progression of Lineage Segregations during Mammalian Embryogenesis by Clonal Analysis. *Dev. Cell* 17, 365–376. doi:10.1016/j.devcel.2009.08.002.

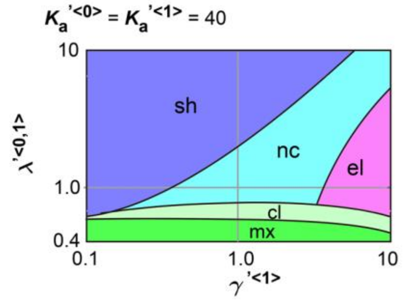

Supplementary Figure S5 (related to Fig. 5): Difference in coefficient of friction causes cell cluster elongation on an expanding field.

Simulations were performed and a phase diagram was generated in a similar manner to Fig. 5, except for different area elasticity values: $K_a'^{<0>} = K_a'^{<1>} = 40$. The phase diagram shows a nearly identical pattern to Fig. 5A.

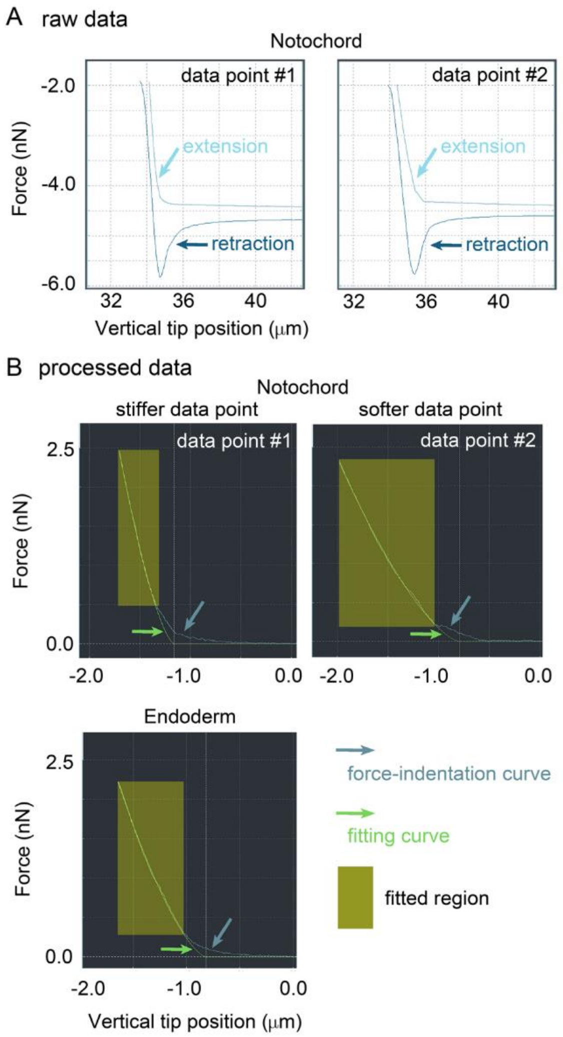

Supplementary Figure S6 (related to Fig. 8): Examples of force–indentation curves in AFM measurements are shown.
A. A force–indentation curve is illustrated in Fig. 8C, and here, a few raw data of force–indentation curves are exemplified. The curves at two data points on the notochord region are shown. The two lines for each panel represent cantilever extension (light blue) and retraction (dark blue).
B. A few processed data of force–indentation curves are exemplified which include two data points in the notochord and one data point in the endoderm. The two data points in the notochord correspond to those in A (data point #1 and #2). In addition to the force–indentation curves (light blue lines), fitting curves used for calculating the Young's moduli are shown (green lines). The values of x– and y–axes were modified from A during the data processing. We considered that embryonic surfaces are located around the right side of the negative peaks of the retraction curves in A, and the corresponding positions were roughly set at x = -1.0~0.0 μm in B. Around the surfaces, probably due to the micro structures of the tissues, the force–indentation curves were usually undulated, and thus, these regions were omitted from the calculations of the Young's moduli. The fitted regions are also shown (dark yellow). In the left panel of the notochord, the slope in the fitted region is steeper than that in the right panel, which should yield a larger value of the Young's modulus. Therefore, the tissue in the left panel is stiffer than that in the right panel.
